# Revascularization of chronic occluded celiac artery for gastroduodenal coil embolization in massive upper gastrointestinal bleed

**DOI:** 10.1016/j.jvscit.2025.101750

**Published:** 2025-02-11

**Authors:** Esther H. Shim, Ryan Lydon, Stephanie S. Hyon, Robert J. Plummer, Thomas Y. Lee, Hal Ginsberg

**Affiliations:** a Department of Surgery, Morristown Medical Center, Morristown, NJ; b Department of Vascular Surgery, Rochester Regional Health, Rochester, NJ; c Department of Vascular Surgery, Morristown Medical Center, Morristown, NJ; d Department of Interventional Radiology, Morristown Medical Center, Morristown, NJ

**Keywords:** Celiac artery occlusion, Gastroduodenal artery embolization, Upper gastrointestinal bleeding (UGIB), Endovascular revascularization, Peptic ulcer disease (PUD), Atherosclerotic disease

## Abstract

Upper gastrointestinal bleeding is a serious condition often linked to peptic ulcer disease, contributing to significant morbidity and mortality. A 78-year-old male presented with upper gastrointestinal bleeding that required blood product transfusions despite multiple endoscopic interventions. Although embolization or surgical ligation of the gastroduodenal artery was considered, angiography revealed celiac trunk occlusion, which would increase the risk of hepatic ischemia. Recanalization and stenting of the celiac trunk was performed, facilitating successful embolization of the gastroduodenal artery. This case illustrates the importance of considering anatomical variations and patient risk factors for visceral arterial occlusions, reducing morbidity and mortality.

Peptic ulcer disease (PUD) is characterized by erosion of the inner lining of the gastrointestinal (GI) tract due to excessive gastric acid secretion, leading to ulcer formation in the stomach and proximal duodenum. Although the prognosis is favorable with appropriate treatment of the underlying cause, recurrence is common, with rates exceeding 60%,^1^ and rebleeding rates ranging from 10% to 24% despite endoscopic management.^2^ For recurrent or persistent bleeding despite endoscopic interventions, embolization of the gastroduodenal artery (GDA) may be indicated.^3^ Surgical intervention may be indicated for those who continue to have stigmata of bleeding despite embolization or those who are unable to undergo angiographic intervention. However, surgical intervention carries significant risk of morbidity and mortality.^4^^,^^5^^,^^6^ The GDA primarily supplies blood to the pylorus, proximal duodenum, and head of the pancreas, along with serving as the terminal branch of both the common hepatic artery and proper hepatic artery. Although embolization of the GDA is usually well-tolerated, concomitant occlusion of the celiac trunk eliminates collateral circulation, which increases the risk of hepatic ischemia. Occlusions of the celiac artery or superior or inferior mesenteric artery are often caused by atherosclerotic disease, with common risk factors including smoking, hypertension, dyslipidemia, and advanced age.^7^ These factors should be considered in patients presenting with an upper gastrointestinal bleed (UGIB) that requires intervention. In this context, we present a successful case of endovascular control of UGIB through recanalization of an occluded celiac artery allowing for coil embolization of the GDA.

## Case report

**Fig 1:**
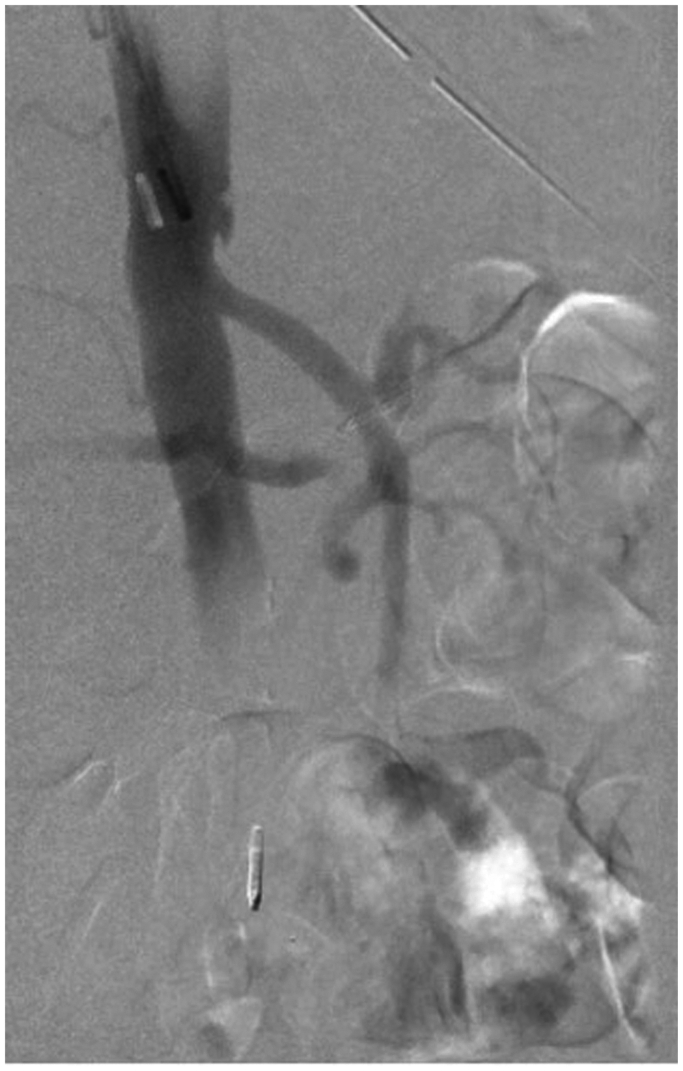
Occluded celiac artery with superior mesenteric artery (SMA) takeoff and bilateral renal arteries inferiorly.

**Fig 2:**
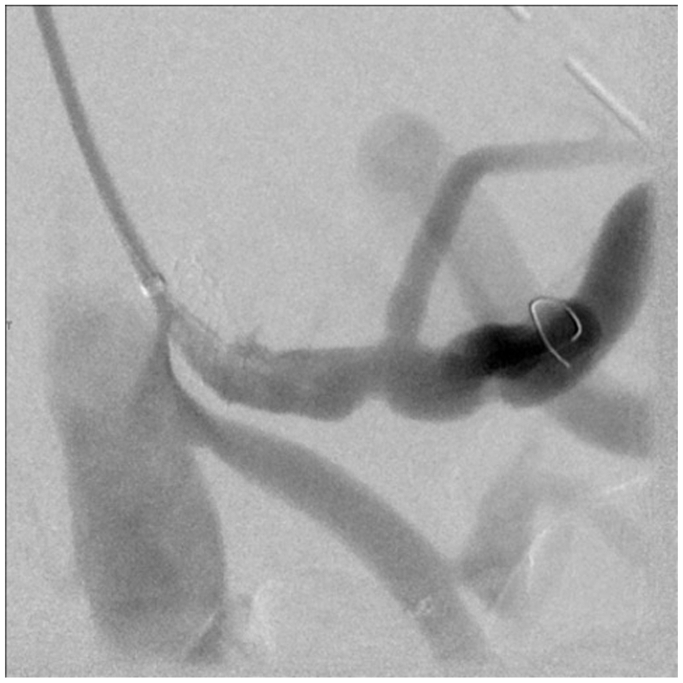
Selective catheterization and stenting of the celiac artery via a left brachial artery approach.

**Fig 3:**
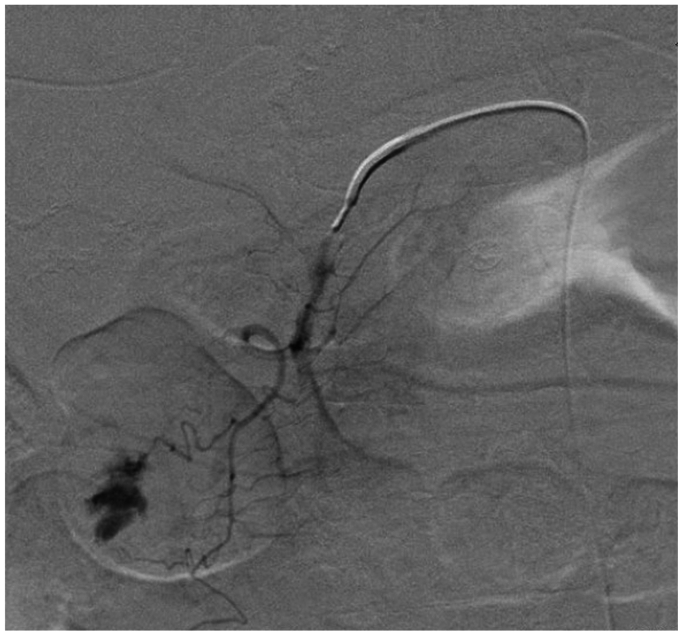
Catheter advancement to the proper hepatic artery into the gastroduodenal artery (GDA), injected contrast demonstrating active hemorrhage from branch of GDA.

**Fig 4:**
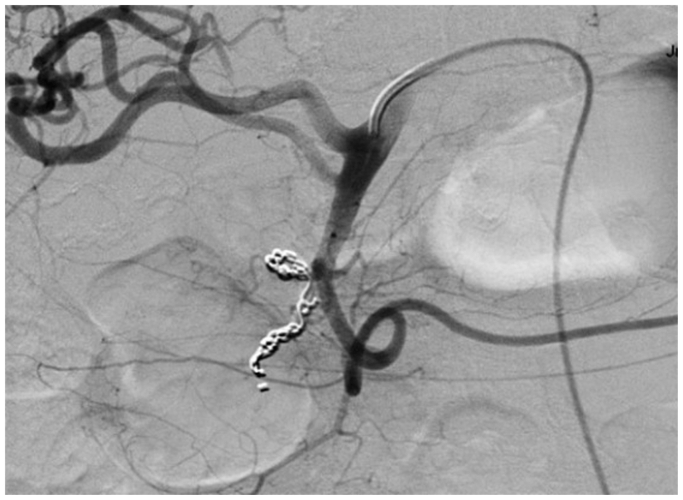
Successful embolization of an actively bleeding supraduodenal branch of the gastroduodenal artery (GDA) using multiple microcoils, with an aberrant left hepatic artery arising from the proximal GDA.

**Fig 5:**
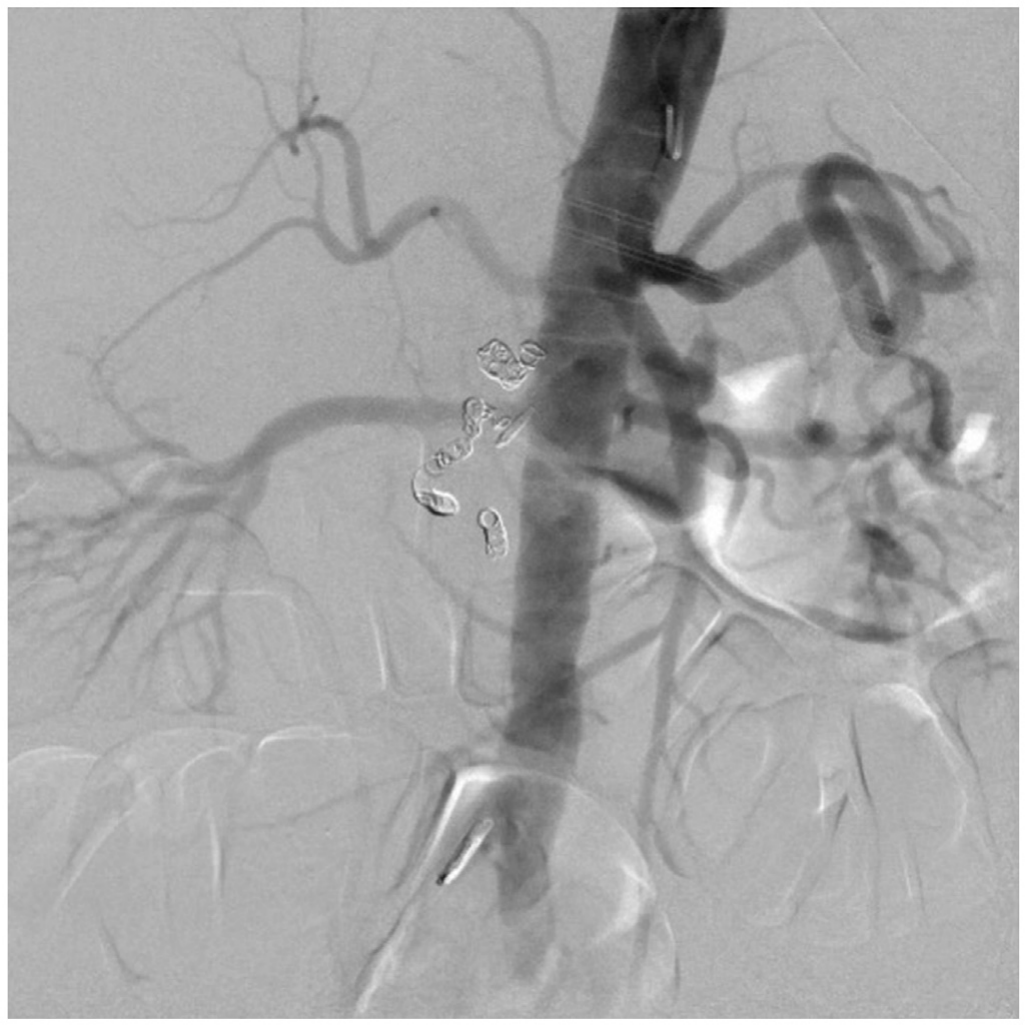
Completion angiogram with revascularization of celiac axis.

A 78-year-old male with a history of myocardial infarction, chronic systolic heart failure, hypertension, 50-pack-year smoking history, stage I diffuse large B-cell lymphoma on chemotherapy, and duodenal ulcers presented to our institution with multiple episodes of melena on the day prior to admission. The patient was initially admitted with concerns for febrile neutropenia following chemotherapy but developed persistent episodes of significant melena. He was subsequently transferred to the medical intensive care unit for resuscitation and required multiple blood product transfusions, including eight units of packed red blood cells, four units of fresh frozen plasma, and two units of platelets. After stabilization on hospital day 6, he underwent an endoscopy notable for a bleeding duodenal ulcer with a visible vessel, which was injected with epinephrine and successfully cauterized with bipolar probe. However, the patient continued to have episodes of bleeding and blood transfusion requirements warranting evaluation by interventional radiology for possible GDA embolization. Computed tomography imaging was deferred given the patient’s variable hemodynamic instability. During his angiography on hospital day 8, the celiac artery was noted to be occluded (Fig 1), and the GDA was found to be enlarged with retrograde filling into the hepatic system. An active bleed was not visualized, and empiric embolization of the GDA was not pursued, given the risk of hepatic necrosis. Over the next several hours, the patient continued to bleed with periods of hemodynamic instability despite massive transfusion. Surgical consultations were sought from General and Vascular Surgery for potential oversewing of the GDA and consideration of a possible celiac artery bypass; however, both options were high risk due to concerns of hepatic ischemia from oversewing the GDA as well as instability of the patient to undergo a celiac artery bypass. Thus, the decision was made to proceed with a combined endovascular approach involving both Interventional Radiology and Vascular Surgery on hospital day 9. Using a left brachial artery approach, and a 6 French, 70 cm Ansel sheath (Cook Medical) followed by a multipurpose catheter (Angiodynamics) and standard angled glidewire (Terumo) were used to selectively catheterize the celiac artery. After successfully traversing the occlusion, the wire was exchanged out for an SV-5 wire (Cordis), and the celiac artery was stented (Fig 2) with a 7 mm × 18 mm balloon expandable stent (Cordis). The stent was then post-plastied with a 9-mm balloon (Bard) as the artery was measured angiographically to be 9 mm. An angiogram at this point revealed a patent celiac axis with return of native arterial flow. An 0.018 guidewire, followed by a 5 French Cobra catheter (Boston Scientific) was used to selectively catheterize the celiac axis and advanced into the proper hepatic artery into the GDA. Contrast injection demonstrated active hemorrhage from a branch of the GDA (Fig 3). A microcatheter (Terumo) was initially used to selectively catheterize the supraduodenal branch of the GDA, but attempts were unsuccessful. The decision was then made to access the bleeding vessel via the superior mesenteric artery (SMA). An angled glide catheter (Terumo) along with a standard angled glidewire was used to selectively catheterize the GDA, facilitating access to the GDA from the SMA. A slight blush was noted at this point near the previously placed endoscopic clips. Multiple coils (Cook Nester) were introduced into the vessel both on the celiac artery and SMA portion of the GDA (Fig 4), as the GDA was unable to be traversed via the celiac route. Completion angiogram revealed patency of the celiac and SMA systems with no flow in the GDA (Fig 5). There were no further signs of bleeding post-procedure, and the patient was placed on daily aspirin 81 mg for 3 months. Written informed consent was obtained from the patient for publication of this case report and accompanying images.

## Discussion

Despite improvements in the medical management of peptic ulcer disease, massive UGIB continues to be a serious problem worldwide, with significant morbidity and mortality.^8^ Peptic ulcer disease is the leading cause of upper GI bleeding, resulting in 150,000 hospital admissions annually.^9^ Endoscopic therapy by gastroenterology has become the primary treatment modality, with approximately 80% of patients having complete cessation of bleeding after one endoscopic procedure.^2^ Although it seems clear that repeat endoscopy is the most effective treatment modality in these patients, there is still a small percentage of patients whose bleeding cannot be controlled endoscopically, and more advanced treatment is required.^10^ Nowadays, endovascular techniques such as transcatheter coil embolization have become the next line of defense, whereas surgical intervention has become the last resort due to the significant associated morbidity and mortality, particularly when associated with PUD.^11^

Aberrant anatomy along with flow abnormalities from atherosclerotic disease can cause challenges for intervening physicians, increasing risks of unintended ischemia. Preoperative arteriography in patients undergoing pancreaticoduodenectomy has identified celiac artery occlusion in up to 10% of cases,^12^^,^^13^ necessitating critical test clamping of the GDA prior to its division to ensure adequate hepatic blood flow. In this context, given the lack of collateral arterial supply, both surgical ligation and angiographic embolization of the GDA—particularly with an aberrant left hepatic artery arising from the GDA—would have posed a significant risk of hepatic ischemia without revascularization of the celiac artery. Several cases have documented hepatic necrosis in patients with aberrant hepatic artery anatomy, emphasizing the importance of recognizing these variations. A retrospective study involving 5625 patients found that 16.32% had an aberrant left hepatic artery, whereas 15.63% exhibited an aberrant right hepatic artery.^14^ These findings demonstrate the relatively high prevalence of such anatomical variations, which can significantly affect surgical and interventional procedures. For example, one case report described complications following embolization of the left gastric artery, resulting in gastric and hepatic infarction due to a replaced left hepatic artery.^15^ Another study documented two cases of postoperative hepatic infarction caused by unintentional ligation of variant hepatic arteries.^16^ The potential consequences of these anatomical variations stress the need for preoperative and intraoperative identification to prevent serious complications and improve patient outcomes. Fortunately, advancements in endovascular techniques have made revascularization of both the celiac and SMA common practice, with widely published cases of successful outcomes.^17^^,^^18^

As illustrated, the combination of endovascular stenting and embolization offers a minimally invasive approach for managing GI bleeding in patients at risk of celiac artery occlusion. This also highlights the importance of accounting for the patient’s medical history and recognizing risk factors for atherosclerosis, which are crucial to identifying individuals more susceptible to visceral artery occlusions who may benefit from more specialized approaches. Other potential etiologies for celiac artery occlusion, such as embolism, median arcuate ligament syndrome (MALS), and dissection, should also be considered. Embolism is unlikely in this case due to the absence of atrial fibrillation, cardiac arrhythmias, or ischemic events, although the patient’s history of malignancy and potential coagulopathy may still warrant further evaluation. MALS remains a possibility, particularly given the post-stenotic dilation and increased size of the celiac artery following the occlusion. Although there is no known history of postprandial pain or unintentional weight loss, such symptoms could have made MALS a more likely diagnosis. Dissection, which can occur spontaneously, particularly in older patients or those with predisposing factors such as hypertension or connective tissue disorders, might also have been a contributing factor. Although the patient has no history of trauma or connective tissue disorders, the abrupt occlusion seen on angiography does raise suspicion for dissection as a potential cause. However, given the patient’s significant risk factors, including coronary artery disease, myocardial infarction, and a history of smoking, atherosclerosis is most likely the primary contributing factor to the celiac artery occlusion. With an ever-aging population that is becoming more medically complex, hybrid endovascular approaches are expected to become more widely utilized. This case emphasizes the need for a comprehensive assessment of both anatomic features and patient characteristics in developing effective treatment strategies, particularly when applying minimally invasive methods to manage GI bleeds. Integrating patient-specific risk factors into the management algorithm will be crucial to optimizing outcomes in such cases.

## Funding

None.

## Disclosures

None.
